# Extended Study of *NUS1* Gene Variants in Parkinson's Disease

**DOI:** 10.3389/fneur.2020.583182

**Published:** 2020-10-27

**Authors:** Lamei Yuan, Xiangyu Chen, Zhi Song, Weidong Le, Wen Zheng, Xin Liu, Hao Deng

**Affiliations:** ^1^Center for Experimental Medicine, The Third Xiangya Hospital, Central South University, Changsha, China; ^2^Department of Neurology, The Third Xiangya Hospital, Central South University, Changsha, China; ^3^Center for Clinical Research on Neurological Diseases, The First Affiliated Hospital, Dalian Medical University, Dalian, China

**Keywords:** NUS1, Parkinson's disease, genetic analysis, whole exome sequencing, whole genome sequencing

## Abstract

Parkinson's disease (PD), is the second most common neurodegenerative disorder worldwide. Genetic, environmental factors, and aging are its primary development contributors. Recently the nuclear undecaprenyl pyrophosphate synthase 1 homolog (*Saccharomyces cerevisiae*) gene (*NUS1*) was reported as a candidate gene for PD, which raised our interest in the relationship between *NUS1* and PD. This study was aimed to further explore the role of *NUS1* variants in PD development. Genetic analysis for 308 Han-Chinese PD patients and 308 ethnically matched controls using whole exome sequencing was conducted. Additionally, a total of 60 articles involving in whole exome/whole genome sequencing or direct sequencing of the *NUS1* gene from PubMed database between July 1, 2011 and August 26, 2020 were reviewed to evaluate PD-associated *NUS1* variants. No potentially pathogenic *NUS1* variant was found in 308 PD cases, and no frequency biases between 308 PD cases and 308 controls were observed for the only non-synonymous variant p.Asp179Glu (genotype: χ^2^ = 0.093, *P* = 0.761; allele: χ^2^ = 0.092, *P* = 0.762). No pathogenic or disease-associated *NUS1* variant was reported in the 5,636 PD cases of the 60 articles. In summary, current findings indicate that *NUS1* variant is not a common genetic factor contributing to PD.

## Introduction

Parkinson's disease (PD), initially named “paralysis agitans” by James Parkinson in 1817, is the second most prevalent neurodegenerative disorder with an incidence of 10–18 per 100,000 individuals worldwide yearly (1, 2). This common disorder affects 1% of total people over 60 and ~4–5% of total people at 85 or older (3, 4). The typical PD pathological characteristics are loss of dopaminergic neurons within the substantia nigra, α-synuclein accumulation in the Lewy bodies, and neuroinflammation (2). It is a complex multifactorial neurodegenerative disorder, in which environmental, genetic factors and increasing age contribute to its development (5), and has a heterogeneous disease progression characterized by a set of clinical features, including bradykinesia, rest tremor, rigidity, postural instability, and a good response to dopaminergic treatment, as well as other non-motor manifestations (2, 6–9). Age has been reported as the single greatest risk factor for sporadic PD (3, 4), while conservative heritability estimates due to common PD genetic risk are about 30% (10).

Revolutionary technologies, especially second-generation sequencing, have expedited the discovery of various PD-associated genes (11). Causative mutations and susceptibility variants are involved in PD pathogenesis by resulting in multiple cellular processes dysfunction, including synaptic function, protein aggregates, intracellular trafficking, ubiquitin-proteasome system, mitochondrial function, autophagy lysosomal pathway, neurite structure, and prion-like transmission (2, 12). In addition to mutations causing monogenic PD which accounts for <5–10% of all cases, the list of susceptibility variants contributing to the development of PD in sporadic cases is steadily increasing (12–15).

The nuclear undecaprenyl pyrophosphate synthase 1 homolog (*S. cerevisiae*) gene (*NUS1*) was recently proposed as a candidate disease-causing gene for PD (16), which raised our interest in potential association between the *NUS1* gene variants and PD. Genetic analysis was conducted in Han-Chinese PD patients and controls from mainland China to further explore the role of *NUS1* gene variants in PD.

## Methods

### Subjects and Clinical Assessments

Sequencing data for *NUS1* coding regions and exon-intron boundaries were extracted from an in-house PD-control exome database. The database contained exome sequencing data for 308 Han-Chinese patients clinically diagnosed with PD [male/female: 154/154, 216 unrelated sporadic cases and 92 probands with familial PD (at least 1 PD-affected family member in 3 generations)] and 308 ethnically matched controls (male/female: 154/154, mean age 58.07 ± 13.59 years) with no signs or family history of PD or similar disorders. All patients enrolled in the study were clinically assessed and diagnosed with PD based on the International Parkinson and Movement Disorder Society (MDS) clinical diagnostic criteria by two independent neurologists from the Third Xiangya Hospital, Central South University (9). The mean age of PD patients was 58.55 ± 11.81 years and the mean age at disease onset was 54.71 ± 12.65 years [33 early onset PD (EOPD, onset age ≤ 40 years old)] (17). The study received the approval from the Institutional Review Board of the Third Xiangya Hospital of Central South University, China, and informed consents about using peripheral blood samples and clinical information for research and publication were signed by all participants.

### Genetic Analysis

The standard phenol-chloroform extraction procedure was used to extract genomic DNA (gDNA) from peripheral blood leukocytes (18, 19). The genetic analysis was performed using whole exome sequencing. Whole exome sequencing was conducted using the extracted gDNA to construct an in-house PD-control exome database. All variants, including single nucleotide polymorphisms (SNPs) and insertions-deletions (InDels), were further annotated with population databases including the 1,000 Genomes Project, the Exome Aggregation Consortium (ExAC), and the Genome Aggregation Database (gnomAD) (20). Variant's potential impact on protein structure or function was predicted by bioinformatic tools including MutationTaster, Sorting Intolerant from Tolerant (SIFT), Protein Variation Effect Analyzer (PROVEAN), and Protein Analysis Through Evolutionary Relationships (PANTHER), and Polymorphism Phenotyping version 2 (PolyPhen-2) (21). Sanger sequencing was applied to further *NUS1* variant confirmation using the following primers: 5′-GCAAGAACTTCTGGGCCT-3′ and 5′-AGAGTAACAGAGCAACGTGAA-3′.

### Statistical Analysis and PubMed Database Screening

Hardy–Weinberg equilibrium was applied to estimate the normal deviation of genotypes in 308 controls. Genotypic and allelic frequencies were calculated, and Pearson's χ^2^ tests were completed for testing significances of genotypic and allelic frequency differences between the 308 PD patients and 308 controls, in which a two-tailed *P* <0.05 was considered statistically significant (22, 23). All statistical analysis was performed using Predictive Analytics Software Statistics 18.0 (SPSS Inc., Chicago, IL). Whole exome/whole genome sequencing studies aimed to identify the disease-causing and/or susceptibility variants for PD were screened through PubMed database using the following search terms: “exome sequencing” or “whole genome sequencing” and “Parkinson's disease”. In addition, a PubMed search using search terms “NUS1” and “Parkinson's disease” was also performed to screen genetic study of the *NUS1* gene in PD patients. These studies were included for searching PD-associated *NUS1* variants.

## Results

No potentially pathogenic *NUS1* variant was found in 308 PD cases. Only a known single-nucleotide *NUS1* non-synonymous variant (c.537T>A, p.Asp179Glu, rs28362519) was detected in 5 PD patients and 6 controls, and confirmed by Sanger sequencing. It was recorded in 1,000 Genomes Project, ExAC, and gnomAD with relative high allele frequencies. This variant was predicted to be disease causing by MutationTaster, while SIFT, PROVEAN, PANTHER, and PolyPhen-2 predicted it had no impact on protein structure or function (Table 1). Genotypic distributions of the rs28362519 variant complied with Hardy–Weinberg equilibrium in the control group (*P* > 0.05). No frequency biases between 308 PD cases and 308 controls were observed for this variant (genotype: χ^2^ = 0.093, *P* = 0.761; allele: χ^2^ = 0.092, *P* = 0.762; Table 2). No pathogenic or disease-associated *NUS1* variants in the 5,142 PD cases containing at least 116 EOPD was reported in the 59 articles retrieved in PubMed database between July 1, 2011 and August 26, 2020 (Supplementary Table 1). Additionally, a genetic study also showed a negative result in 494 sporadic PD cases by direct sequencing of full coding regions and exon-intron boundaries of the *NUS1* gene (24).

**Table 1:** Allele frequencies and bioinformatic predictions of the *NUS1* c.537T>A variant.

**Variant**		**Zygosity**	**dbSNP154 rs number**	**Allele frequencies**			**Bioinformatic predictions**				
**Nucleotide change**	**Amino acid change**			**1,000 Genomes Project**	**ExAC**	**gnomAD**	**MutationTaster**	**SIFT**	**PROVEAN**	**PANTHER**	**PolyPhen-2**
c.537T>A	p.Asp179Glu	Heterozygote	rs28362519	2 × 10^−3^	6.802 × 10^−4^	6.824 × 10^−4^	Disease causing	Tolerated	Neutral	Probably benign	Benign

*dbSNP154, Single Nucleotide Polymorphism Database version 154; rs, Reference SNP; ExAC, Exome Aggregation Consortium; gnomAD, Genome Aggregation Database; SIFT, Sorting Intolerant from Tolerant; PROVEAN, Protein Variation Effect Analyzer; PANTHER, Protein Analysis Through Evolutionary Relationships; PolyPhen-2, Polymorphism Phenotyping version 2*.

**Table 2:** Genotypic and allelic distributions of rs28362519 in Han-Chinese patients with Parkinson's disease and ethnically matched controls.

**Genotype/Allele**	**Patients (freq)**	**Controls (freq)**	**χ^2^-value**	***P*-value**	**OR (95% CI)**
TT	303 (0.984)	302 (0.981)	0.093	0.761	0.831 (0.251–2.751)
TA	5 (0.016)	6 (0.019)			
AA	0	0			
T	611 (0.992)	610 (0.990)	0.092	0.762	0.832 (0.253–2.741)
A	5 (0.008)	6 (0.010)			

*Freq, frequency; OR, odds ratio; CI, confidence interval*.

## Discussion

PD is a neurological disorder arising from a complex interplay among genetic, environmental cues, and aging (16, 25). The synuclein alpha gene (*SNCA*) was discovered as the first causative gene for PD in 1997 (3, 26), and since then, dramatic progress has been made in identification of disease-causing gene for Mendelian PD (27, 28). As of this writing, at least 23 disease-causing loci and 19 genes have been identified to be responsible for monogenic PD form which explained <10% of all cases (12, 13). Susceptibility variants in certain genes, such as the glucosylceramidase beta (*GBA*), microtubule associated protein tau (*MAPT*), *SNCA*, and leucine rich repeat kinase 2 (*LRRK2*) genes, have also been reported to increase PD risk (8, 12).

Intriguingly, variants in the *NUS1* gene were reported to be responsible for PD in Chinese patients (16). The *NUS1* gene, mapped to chromosome 6q22.1, encodes a membrane protein-Nogo-B receptor (NgBR), which is required for the biosynthesis of dolichol and protein glycosylation (29, 30). Homozygous *NUS1* mutations in this gene were previously described to cause congenital disorder of glycosylation type Iaa (CDG1AA, OMIM 617082) (30), while rare heterozygous mutations were reported in two cases with autosomal dominant mental retardation-55 with seizures (MRD55, OMIM 617831) (31).

In this study, exome sequencing data involving coding regions and exon-intron boundaries of *NUS1*, were meticulously evaluated in 308 Han-Chinese patients containing 33 EOPD and 308 ethnically matched controls. Despite great interest, no association was found between the *NUS1* variant p.Asp179Glu and PD phenotype, indicating that variants in the *NUS1* gene may not be a primary genetic contributor to PD. The power analysis involving 5,944 PD cases (308 in this study and 5,636 from 60 articles) and 308 controls revealed that *NUS1* mutations accountable for monogenic PD should affect <0.5% in general PD cases (power = 0.909) as the non-essential genetic contributor.

The findings lure us to hold curiosity about the real impacts of *NUS1* in PD. Moreover, Guo et al.'s study lacked evidence of co-segregation of *NUS1* variants in families (16), which raises concerns whether the *NUS1* gene is indeed a disease-causing gene for monogenic PD. Our suspicion is supported by the following observations: (i) The *de novo* heterozygous *NUS1* c.691+3dupA variant may be related to PD in an individual with PD phenotype (16), but other gene variants causing autosomal recessive parkinsonism should be excluded. In one report 44 to 82 *de novo* single-nucleotide variants occur in an individual genome, and some *de novo* variants, located in regulatory regions, may result in a disease phenotype (32). (ii) If the *de novo NUS1* gene variant indeed caused autosomal dominant PD, it is of interest to discard the priority of screening variants in familial PD cases. Enough familial PD cases could be acquired for verifying the pathogenicity of heterozygous *NUS1* variants, due to 5,089 sporadic PD cases enrolled in Guo et al.'s study and ~10–15% of PD cases having family history (16, 33). (iii) Since *NUS1* variants were found in sporadic PD cases, it is of interest to abandon screening the variants in relatives. All the 26 *NUS1* variant carriers having unavailable relatives and lack of detailed data involving exclusion of all known or unknown PD-causing gene mutations further query the core role of *NUS1* variants in PD. (iv) Mouse models with genetic deficiencies in transcription factor genes, such as the engrailed homeobox 1/2 gene (*En1/2*) and the paired like homeodomain 3 gene (*Pitx3*), exhibited features of PD, especially dopaminergic neuronal loss in the substantia nigra, but no definite pathogenic gene mutations were found in PD patients (34). Moreover, *Drosophila* with mutant spinocerebellar ataxia 3 (SCA3) selectively expressed in dopaminergic neurons, a non-PD causative gene deficiency, also presents PD-like phenotype (35). These challenge the persuasiveness of functional studies in *Drosophila* models (16).

In summary, current findings indicate that *NUS1* variant is not a common genetic contributor to PD. However, some limitations to this study should be acknowledged: (i) The possibility that a few of whole exome/whole genome sequencing studies might miss certain *NUS1* variant(s) during sequence capture cannot be excluded. (ii) Some whole exome/whole genome sequencing studies focusing on investigating the disease-causing and/or susceptibility variants for PD in other databases such as the Cochrane and Embase databases may be missed. Further studies involving *NUS1* variants in familial PD, particularly in EOPD, using high-coverage next-generation sequencing, and throughout functional studies in main pathobiological pathways underpinning PD (3), including autophagy, endocytosis, mitochondrial biology, immune response, and lysosomal function, are warranted to expose the real role of the *NUS1* gene in PD development.

## Data Availability Statement

The raw data supporting the conclusions of this article will be made available by the authors, without undue reservation.

## Ethics Statement

The study involving human participants was reviewed and approved by the Institutional Review Board of the Third Xiangya Hospital of Central South University in Changsha, Hunan, China (approval number: 2018-S400). The patients/participants provided their written informed consent to participate in this study.

## Author Contributions

HD and LY conceived and designed this study. ZS and WZ collected the patient samples and clinical data. XC and XL performed the experiments. WL and HD analyzed the data. XC and LY wrote the manuscript. All authors read and approved the final version of the manuscript.

## Conflict of Interest

The authors declare that the research was conducted in the absence of any commercial or financial relationships that could be construed as a potential conflict of interest.
