# Supplementary material for: Extended Study of NUS1 Gene Variants in Parkinson's Disease
Source: Front Neurol. 2020 Oct 27;11:583182. doi: 10.3389/fneur.2020.583182 (PMC7653662; doi:10.3389/fneur.2020.583182)
Supplement: Supplementary file 1 [file Table_1.doc]

**Supplementary Table 1.** Published reports of genetic analysis in patients with Parkinson’s disease by whole exome/whole genome sequencing

| Reference* | Country/Population | Number of patients |
| --- | --- | --- |
| Vilariño-Güell et al. (1)^†^ | Swiss | 2 |
| Zimprich et al. (2)^†^ | Austrian | 2 |
| Edvardson et al. (3)^†^ | Palestinian | 1 |
| Köroğlu et al. (4)^†^ | NA | 1 |
| Lesage et al. (5)^†^ | France | 3 |
| Krebs et al. (6)^†^ | Iran | 2 |
| Quadri et al. (7)^†^ | Sicily of Italy | 2 |
| Schulte et al. (8)^†^ | Germany | 2 |
| Schulte et al. (9)^†^ | Germany | 2 |
| Vilariño-Güell et al. (10)^†^ | Canada | 3 |
| Unal Gulsuner et al. (11)^†^ | Turkey | 2 |
| Quadri et al. (12)^†^ | Sardinia of Italy | 100 |
| Lohmann et al. (13)^†^ | Turkey | 2 |
| Yang et al. (14)^†^ | Chinese | 2 |
| Mata et al. (15)^†^ | European | 4 |
| Ruiz-Martínez (16)^†^ | Spanish | 2 |
| Simón-Sánchez et al. (17)^†^ | NA | 1189 |
| Kun-Rodrigues et al. (18)^†^ | European origin | 21 |
| Farlow et al. (19)^†^ | Non-Hispanic European American, Hispanic descent, non-Hispanic Asian, and Middle Eastern descent | 142 |
| Shi et al. (20)^†^ | Chinese from mainland China | 2 |
| Lesage et al. (21)^†^ | European, North African, Turkish, and Lebanese | 62 |
| Chen et al. (22)^†^ | Chinese from mainland China | 1 |
| Deng et al. (23)^†^ | North America and mainland China | 13 |
| Jaberi et al. (24)^†^ | Iranian | 1 |
| Sudhaman et al. (25)^†^ | India | 2 |
| Hanagasi et al. (26)^#^ | Turkish | 1 |
| Kirola et al. (27)^†^ | India | 2 |
| Shi et al. (28)^†^ | Chinese | 2 |
| Sandor et al. (29)^†^ | Thames valley population from UK | 228 |
| Jansen et al. (30)^†^ | European origin | 1148 |
| Siitonen et al. (31)^†^ | Finland | 385 |
| Ruiz-Martínez et al. (32)^†^ | Spain | 4 |
| Butcher et al. (33)^#^ | European descent | 3 |
| Khodadadi et al. (34)^#^ | Iranian | 2 |
| Jansen et al. (35)^†^ | European origin | 1167 |
| Shulskaya et al. (36)^†^ | Russian | 48 |
| Taghavi et al. (37)^#^ | Iranian | 6 |
| Suleiman et al. (38)^†^ | NA | 1 |
| Lee et al. (39)^†^ | Caucasian | 1 |
| Kuipers et al. (40)^†^ | South African Xhosa | 2 |
| Bravo et al. (41)^#^ | NA | 2 |
| Trinh et al. (42)^†^ | Germany | 50 |
| Stephenson et al. (43)^†^ | Jewish of Iranian origin | 2 |
| Yemni et al. (44)^†^ | Saudi Arabia | 60 |
| Lin et al. (45)^†^ | Taiwan in China | 3 |
| Hong et al. (46)^†^ | NA | 1 |
| Milanowski et al. (47)^†^ | Polish origin | 1 |
| Xie et al. (48)^#^ | Chinese | 1 |
| Berenguer-Escuder et al. (49)^†^ | German origin | 2 |
| Gialluisi et al. (50)^†^ | Mainland Italy | 123 |
| Oji et al. (51)^†^ | Japanese | 6 |
| Li et al. (52)^†^ | Chinese from mainland China | 240 |
| Martinelli et al. (53)^†^ | NA | 1 |
| Jin et al. (54)^†^ | Chinese | 2 |
| Martin et al. (55)^†#^ | Belgium | 52 |
| Chen et al. (56)^†^ | Chinese | 21 |
| Li et al. (57)^†^ | Chinese from mainland China | 2 |
| Odumpatta et al. (58)^†^ | South African origin | 7 |
| Gatto et al. (59)^†^ | Argentinian | 1 |
| Total |  | 5142 |

*Articles retrieved in PubMed database from July 1, 2011 to August 26, 2020.

^†^whole exome sequencing study; ^#^whole genome sequencing study.

NA, not available.

**REFERENCES**

1. Vilariño-Güell C, Wider C, Ross OA, Dachsel JC, Kachergus JM, Lincoln SJ, et al. VPS35 mutations in Parkinson disease. *Am J Hum Genet*. (2011) 89**:**162-7. doi:10.1016/j.ajhg.2011.06.001

2. Zimprich A, Benet-Pagès A, Struhal W, Graf E, Eck SH, Offman MN, et al. A mutation in VPS35, encoding a subunit of the retromer complex, causes late-onset Parkinson disease. *Am J Hum Genet*. (2011) 89**:**168-75. doi:10.1016/j.ajhg.2011.06.008

3. Edvardson S, Cinnamon Y, Ta-Shma A, Shaag A, Yim YI, Zenvirt S, et al. A deleterious mutation in DNAJC6 encoding the neuronal-specific clathrin-uncoating co-chaperone auxilin, is associated with juvenile parkinsonism. *PLoS One*. (2012) 7**:**e36458. doi:10.1371/journal.pone.0036458

4. Köroğlu Ç, Baysal L, Cetinkaya M, Karasoy H, Tolun A. DNAJC6 is responsible for juvenile parkinsonism with phenotypic variability. *Parkinsonism Relat Disord*. (2013) 19**:**320-4. doi:10.1016/j.parkreldis.2012.11.006

5. Lesage S, Anheim M, Letournel F, Bousset L, Honoré A, Rozas N, et al. G51D alpha-synuclein mutation causes a novel parkinsonian-pyramidal syndrome. *Ann Neurol*. (2013) 73**:**459-71. doi:10.1002/ana.23894

6. Krebs CE, Karkheiran S, Powell JC, Cao M, Makarov V, Darvish H, et al. The Sac1 domain of SYNJ1 identified mutated in a family with early-onset progressive Parkinsonism with generalized seizures. *Hum Mutat*. (2013) 34**:**1200-7. doi:10.1002/humu.22372

7. Quadri M, Fang M, Picillo M, Olgiati S, Breedveld GJ, Graafland J, et al. Mutation in the SYNJ1 gene associated with autosomal recessive, early-onset Parkinsonism. *Hum Mutat*. (2013) 34**:**1208-15. doi:10.1002/humu.22373

8. Schulte EC, Stahl I, Czamara D, Ellwanger DC, Eck S, Graf E, et al. Rare variants in PLXNA4 and Parkinson's disease. *PLoS One*. (2013) 8**:**e79145. doi:10.1371/journal.pone.0079145

9. Schulte EC, Ellwanger DC, Dihanich S, Manzoni C, Stangl K, Schormair B, et al. Rare variants in LRRK1 and Parkinson's disease. *Neurogenetics*. (2014) 15**:**49-57. doi:10.1007/s10048-013-0383-8

10. Vilariño-Güell C, Rajput A, Milnerwood AJ, Shah B, Szu-Tu C, Trinh J, et al. DNAJC13 mutations in Parkinson disease. *Hum Mol Genet*. (2014) 23**:**1794-801. doi:10.1093/hmg/ddt570

11. Unal Gulsuner H, Gulsuner S, Mercan FN, Onat OE, Walsh T, Shahin H, et al. Mitochondrial serine protease HTRA2 p.G399S in a kindred with essential tremor and Parkinson disease. *Proc Natl Acad Sci U S A*. (2014) 111**:**18285-90. doi:10.1073/pnas.1419581111

12. Quadri M, Yang X, Cossu G, Olgiati S, Saddi VM, Breedveld GJ, et al. An exome study of Parkinson's disease in Sardinia, a Mediterranean genetic isolate. *Neurogenetics*. (2015) 16**:**55-64. doi:10.1007/s10048-014-0425-x

13. Lohmann E, Coquel AS, Honoré A, Gurvit H, Hanagasi H, Emre M, et al. A new F-box protein 7 gene mutation causing typical Parkinson's disease. *Mov Disord*. (2015) 30**:**1130-3. doi:10.1002/mds.26266

14. Yang Y, Tang BS, Weng L, Li N, Shen L, Wang J, et al. Genetic identification is critical for the diagnosis of parkinsonism: a Chinese pedigree with early onset of parkinsonism. *PLoS One*. (2015) 10**:**e0136245. doi:10.1371/journal.pone.0136245

15. Mata IF, Jang Y, Kim CH, Hanna DS, Dorschner MO, Samii A, et al. The RAB39B p.G192R mutation causes X-linked dominant Parkinson's disease. *Mol Neurodegener*. (2015) 10**:**50. doi:10.1186/s13024-015-0045-4

16. Ruiz-Martínez J, Krebs CE, Makarov V, Gorostidi A, Martí-Massó JF, Paisán-Ruiz C. GIGYF2 mutation in late-onset Parkinson's disease with cognitive impairment. *J Hum Genet*. (2015) 60**:**637-40. doi:10.1038/jhg.2015.69

17. Simón-Sánchez J, Heutink P, Gasser T; International Parkinson's Disease Genomics Consortium (IPDGC). Variation in PARK10 is not associated with risk and age at onset of Parkinson's disease in large clinical cohorts. *Neurobiol Aging*. (2015) 36**:**2907.e13-7. doi:10.1016/j.neurobiolaging.2015.07.008

18. Kun-Rodrigues C, Ganos C, Guerreiro R, Schneider SA, Schulte C, Lesage S, et al.; International Parkinson's Disease Genomics Consortium (IPDGC). A systematic screening to identify de novo mutations causing sporadic early-onset Parkinson's disease. *Hum Mol Genet*. (2015) 24**:**6711-20. doi:10.1093/hmg/ddv376

19. Farlow JL, Robak LA, Hetrick K, Bowling K, Boerwinkle E, Coban-Akdemir ZH, et al. Whole-exome sequencing in familial Parkinson disease. *JAMA Neurol*. (2016) 73**:**68-75. doi:10.1001/jamaneurol.2015.3266

20. Shi CH, Mao CY, Zhang SY, Yang J, Song B, Wu P, et al. CHCHD2 gene mutations in familial and sporadic Parkinson's disease. *Neurobiol Aging*. (2016) 38**:**217.e9-13. doi:10.1016/j.neurobiolaging.2015.10.040

21. Lesage S, Drouet V, Majounie E, Deramecourt V, Jacoupy M, Nicolas A, et al. Loss of VPS13C function in autosomal-recessive parkinsonism causes mitochondrial dysfunction and increases PINK1/Parkin-dependent mitophagy. *Am J Hum Genet*. (2016) 98**:**500-13. doi:10.1016/j.ajhg.2016.01.014

22. Chen H, Huang X, Yuan L, Xia H, Xu H, Yang Y, et al. A homozygous parkin p.G284R mutation in a Chinese family with autosomal recessive juvenile parkinsonism. *Neurosci Lett*. (2016) 624**:**100-4. doi:10.1016/j.neulet.2016.05.011

23. Deng HX, Shi Y, Yang Y, Ahmeti KB, Miller N, Huang C, et al. Identification of TMEM230 mutations in familial Parkinson's disease. *Nat Genet*. (2016) 48**:**733-9. doi:10.1038/ng.3589

24. Jaberi E, Rohani M, Shahidi GA, Nafissi S, Arefian E, Soleimani M, et al. Mutation in ADORA1 identified as likely cause of early-onset parkinsonism and cognitive dysfunction. *Mov Disord*. (2016) 31**:**1004-11. doi:10.1002/mds.26627

25. Sudhaman S, Muthane UB, Behari M, Govindappa ST, Juyal RC, Thelma BK. Evidence of mutations in RIC3 acetylcholine receptor chaperone as a novel cause of autosomal-dominant Parkinson's disease with non-motor phenotypes. *J Med Genet*. (2016) 53**:**559-66. doi:10.1136/jmedgenet-2015-103616

26. Hanagasi HA, Giri A, Kartal E, Guven G, Bilgiç B, Hauser AK, et al. A novel homozygous DJ1 mutation causes parkinsonism and ALS in a Turkish family. *Parkinsonism Relat Disord*. (2016) 29**:**117-20. doi:10.1016/j.parkreldis.2016.03.001

27. Kirola L, Behari M, Shishir C, Thelma BK. Identification of a novel homozygous mutation Arg459Pro in SYNJ1 gene of an Indian family with autosomal recessive juvenile Parkinsonism. *Parkinsonism Relat Disord*. (2016) 31**:**124-8. doi:10.1016/j.parkreldis.2016.07.014

28. Shi CH, Zhang SY, Yang ZH, Yang J, Shang DD, Mao CY, et al. A novel RAB39B gene mutation in X-linked juvenile parkinsonism with basal ganglia calcification. *Mov Disord*. (2016) 31**:**1905-9. doi:10.1002/mds.26828

29. Sandor C, Honti F, Haerty W, Szewczyk-Krolikowski K, Tomlinson P, Evetts S, et al. Whole-exome sequencing of 228 patients with sporadic Parkinson's disease. *Sci Rep*. (2017) 7**:**41188. doi:10.1038/srep41188

30. Jansen IE, Ye H, Heetveld S, Lechler MC, Michels H, Seinstra RI, et al. Discovery and functional prioritization of Parkinson's disease candidate genes from large-scale whole exome sequencing. *Genome Biol*. (2017) 18**:**22. doi:10.1186/s13059-017-1147-9

31. Siitonen A, Nalls MA, Hernandez D, Gibbs JR, Ding J, Ylikotila P, et al. Genetics of early-onset Parkinson's disease in Finland: exome sequencing and genome-wide association study. *Neurobiol Aging*. (2017) 53**:**195.e7-10. doi:10.1016/j.neurobiolaging.2017.01.019

32. Ruiz-Martínez J, Azcona LJ, Bergareche A, Martí-Massó JF, Paisán-Ruiz C. Whole-exome sequencing associates novel CSMD1 gene mutations with familial Parkinson disease. *Neurol Genet*. (2017) 3**:**e177. doi:10.1212/nxg.0000000000000177

33. Butcher NJ, Merico D, Zarrei M, Ogura L, Marshall CR, Chow EWC, et al. Whole-genome sequencing suggests mechanisms for 22q11.2 deletion-associated Parkinson's disease. *PLoS One*. (2017) 12**:**e0173944. doi:10.1371/journal.pone.0173944

34. Khodadadi H, Azcona LJ, Aghamollaii V, Omrani MD, Garshasbi M, Taghavi S, et al. PTRHD1 (C2orf79) mutations lead to autosomal-recessive intellectual disability and parkinsonism. *Mov Disord*. (2017) 32**:**287-91. doi:10.1002/mds.26824

35. Jansen IE, Gibbs JR, Nalls MA, Price TR, Lubbe S, Van Rooij J, et al. Establishing the role of rare coding variants in known Parkinson's disease risk loci. *Neurobiol Aging*. (2017) 59**:** 220.e11-18. doi:10.1016/j.neurobiolaging.2017.07.009

36. Shulskaya MV, Alieva AK, Vlasov IN, Zyrin VV, Fedotova EY, Abramycheva NY, et al. Whole-exome sequencing in searching for new variants associated with the development of Parkinson's disease. *Front Aging Neurosci*. (2018) 10**:**136. doi:10.3389/fnagi.2018.00136

37. Taghavi S, Chaouni R, Tafakhori A, Azcona LJ, Firouzabadi SG, Omrani MD, et al. A clinical and molecular genetic study of 50 families with autosomal recessive parkinsonism revealed known and novel gene mutations. *Mol Neurobiol*. (2018) 55**:**3477-89. doi:10.1007/s12035-017-0535-1

38. Suleiman J, Hamwi N, El-Hattab AW. ATP13A2 novel mutations causing a rare form of juvenile-onset Parkinson disease. *Brain Dev*. (2018) 40**:**824-6. doi:10.1016/j.braindev.2018.05.017

39. Lee RG, Sedghi M, Salari M, Shearwood AJ, Stentenbach M, Kariminejad A, et al. Early-onset Parkinson disease caused by a mutation in CHCHD2 and mitochondrial dysfunction. *Neurol Genet*. (2018) 4**:**e276. doi:10.1212/nxg.0000000000000276

40. Kuipers DJS, Carr J, Bardien S, Thomas P, Sebate B, Breedveld GJ, et al. PTRHD1 loss-of-function mutation in an African family with juvenile-onset Parkinsonism and intellectual disability. *Mov Disord*. (2018) 33**:**1814-9. doi:10.1002/mds.27501

41. Bravo P, Darvish H, Tafakhori A, Azcona LJ, Johari AH, Jamali F, et al. Molecular characterization of PRKN structural variations identified through whole-genome sequencing. *Mol Genet Genomic Med*. (2018) 6**:**1243-8. doi:10.1002/mgg3.482

42. Trinh J, Lohmann K, Baumann H, Balck A, Borsche M, Brüggemann N, et al.; International Parkinson's Disease Genomics Consortium (IPDGC). Utility and implications of exome sequencing in early-onset Parkinson's disease. *Mov Disord*. (2019) 34**:**133-7. doi:10.1002/mds.27559

43. Stephenson SE, Djaldetti R, Rafehi H, Wilson GR, Gillies G, Bahlo M, et al. Familial early onset Parkinson's disease caused by a homozygous frameshift variant in PARK7: clinical features and literature update. *Parkinsonism Relat Disord*. (2019) 64**:**308-11. doi:10.1016/j.parkreldis.2019.03.013

44. Yemni EA, Monies D, Alkhairallah T, Bohlega S, Abouelhoda M, Magrashi A, et al. Integrated analysis of whole exome sequencing and copy number evaluation in Parkinson's disease. *Sci Rep*. (2019) 9**:**3344. doi:10.1038/s41598-019-40102-x

45. Lin CH, Chen PL, Tai CH, Lin HI, Chen CS, Chen ML, et al. A clinical and genetic study of early-onset and familial parkinsonism in taiwan: an integrated approach combining gene dosage analysis and next-generation sequencing. *Mov Disord*. (2019) 34**:**506-15. doi:10.1002/mds.27633

46. Hong D, Cong L, Zhong S, He Y, Xin L, Gao X, et al. Clonazepam improves the symptoms of two siblings with novel variants in the SYNJ1 gene. *Parkinsonism Relat Disord*. (2019) 62**:**221-5. doi:10.1016/j.parkreldis.2018.11.020

47. Milanowski Ł, Hoffman-Zacharska D, Geremek M, Friedman A, Figura M, Koziorowski D. The matter of significance - has the p.(Glu121Lys) variant of TOR1A gene a pathogenic role in dystonia or Parkinson disease? *J Clin Neurosci*. (2019) 72:501-3. doi:10.1016/j.jocn.2019.12.018

48. Xie F, Chen S, Cen ZD, Chen Y, Yang DH, Wang HT, et al. A novel homozygous SYNJ1 mutation in two siblings with typical Parkinson's disease. *Parkinsonism Relat Disord*. (2019) 69**:**134-7. doi:10.1016/j.parkreldis.2019.11.001

49. Berenguer-Escuder C, Grossmann D, Massart F, Antony P, Burbulla LF, Glaab E, et al. Variants in Miro1 cause alterations of ER-mitochondria contact sites in fibroblasts from Parkinson's disease patients. *J Clin Med*. (2019) 8:2226. doi:10.3390/jcm8122226

50. Gialluisi A, Reccia MG, Tirozzi A, Nutile T, Lombardi A, De Sanctis C, et al.; International Parkinson's Disease Genomic Consortium (IPDGC). Whole exome sequencing study of Parkinson disease and related endophenotypes in the Italian population. *Front Neurol*. (2019) 10**:**1362. doi:10.3389/fneur.2019.01362

51. Oji Y, Hatano T, Ueno SI, Funayama M, Ishikawa KI, Okuzumi A, et al. Variants in saposin D domain of prosaposin gene linked to Parkinson's disease. *Brain*. (2020) 143**:**1190-205. doi:10.1093/brain/awaa064

52. Li N, Wang L, Zhang J, Tan EK, Li J, Peng J, et al. Whole-exome sequencing in early-onset Parkinson's disease among ethnic Chinese. *Neurobiol Aging*. (2020) 90**:**150.e5-11. doi:10.1016/j.neurobiolaging.2019.12.023

53. Martinelli S, Cordeddu V, Galosi S, Lanzo A, Palma E, Pannone L, et al. Co-occurring WARS2 and CHRNA6 mutations in a child with a severe form of infantile parkinsonism. *Parkinsonism Relat Disord*. (2020) 72**:**75-9. doi:10.1016/j.parkreldis.2020.02.003

54. Jin X, An L, Hao S, Liu Q, Zhang Q, Wang X, et al. Compound heterozygous variants of the FBXO7 gene resulting in infantile-onset Parkinsonian-pyramidal syndrome in siblings of a Chinese family. *J Clin Lab Anal*. (2020) 34**:**e23324. doi:10.1002/jcla.23324

55. Martin S, Smolders S, Van Den Haute C, Heeman B, Van Veen S, Crosiers D, et al. Mutated ATP10B increases Parkinson's disease risk by compromising lysosomal glucosylceramide export. *Acta Neuropathol*. (2020) 139**:**1001-24. doi:10.1007/s00401-020-02145-7

56. Chen H, Jin YH, Xue YY, Chen YL, Chen YJ, Tao QQ, et al. Novel ATP13A2 and PINK1 variants identified in Chinese patients with Parkinson's disease by whole-exome sequencing. *Neurosci Lett*. (2020) 733**:**135075. doi:10.1016/j.neulet.2020.135075

57. Li T, Kou D, Cui Y, Le W. Whole exome sequencing identified a new compound heterozygous PRKN mutation in a Chinese family with early-onset Parkinson's disease. *Biosci Rep*. (2020) 40:BSR20200534. doi:10.1042/bsr20200534

58. Odumpatta R, Mohanapriya A. Next generation sequencing exome data analysis aids in the discovery of SNP and INDEL patterns in Parkinson's disease. *Genomics*. (2020) 112**:**3722-8. doi:10.1016/j.ygeno.2020.04.025

59. Gatto EM, Rojas GJ, Nemirovsky SI, Da Prat G, Persi G, Cesarini M, et al. A novel mutation in PSEN1 (p.Arg41Ser) in an Argentinian woman with early onset Parkinsonism. *Parkinsonism Relat Disord*. (2020) 77**:**21-5. doi:10.1016/j.parkreldis.2020.06.005
